# Why ‘the uplift of the Tibetan Plateau’ is a myth

**DOI:** 10.1093/nsr/nwaa091

**Published:** 2020-05-05

**Authors:** Robert A Spicer, Tao Su, Paul J Valdes, Alexander Farnsworth, Fei-Xiang Wu, Gongle Shi, Teresa E V Spicer, Zhekun Zhou

**Affiliations:** CAS Key Laboratory of Tropical Forest Ecology, Xishuangbanna Tropical Botanical Garden, Chinese Academy of Sciences, Mengla 666303, China; Center of Plant Ecology, Core Botanical Gardens, Chinese Academy of Sciences, Mengla 666303, China; School of Environment, Earth and Ecosystem Sciences, The Open University, Milton Keynes MK7 6AA, UK; CAS Key Laboratory of Tropical Forest Ecology, Xishuangbanna Tropical Botanical Garden, Chinese Academy of Sciences, Mengla 666303, China; Center of Plant Ecology, Core Botanical Gardens, Chinese Academy of Sciences, Mengla 666303, China; School of Geographical Sciences, University of Bristol, Bristol BS8 1SS, UK; School of Geographical Sciences, University of Bristol, Bristol BS8 1SS, UK; Key Laboratory of Vertebrate Evolution and Human Origins, Institute of Vertebrate Paleontology and Paleoanthropology, Chinese Academy of Sciences, Beijing 100044, China; State Key Laboratory of Palaeobiology and Stratigraphy, Nanjing Institute of Geology and Palaeontology and Center for Excellence in Life and Paleoenvironment, Chinese Academy of Sciences, Nanjing 210008, China; CAS Key Laboratory of Tropical Forest Ecology, Xishuangbanna Tropical Botanical Garden, Chinese Academy of Sciences, Mengla 666303, China; CAS Key Laboratory of Tropical Forest Ecology, Xishuangbanna Tropical Botanical Garden, Chinese Academy of Sciences, Mengla 666303, China; Center of Plant Ecology, Core Botanical Gardens, Chinese Academy of Sciences, Mengla 666303, China

**Keywords:** Tibet, paleoaltimetry, paleogeography, paleontology, Himalaya

## Abstract

The often-used phrase ‘the uplift of the Tibetan Plateau’ implies a flat-surfaced Tibet rose as a coherent entity, and that uplift was driven entirely by the collision and northward movement of India. Here, we argue that these are misconceptions derived in large part from simplistic geodynamic and climate modeling, as well as proxy misinterpretation. The growth of Tibet was a complex process involving mostly Mesozoic collisions of several Gondwanan terranes with Asia, thickening the crust and generating complex relief before the arrival of India. In this review, Earth system modeling, paleoaltimetry proxies and fossil finds contribute to a new synthetic view of the topographic evolution of Tibet. A notable feature overlooked in previous models of plateau formation was the persistence through much of the Cenozoic of a wide east–west orientated deep central valley, and the formation of a plateau occurred only in the late Neogene through compression and internal sedimentation.

## INTRODUCTION

**Figure 1.**
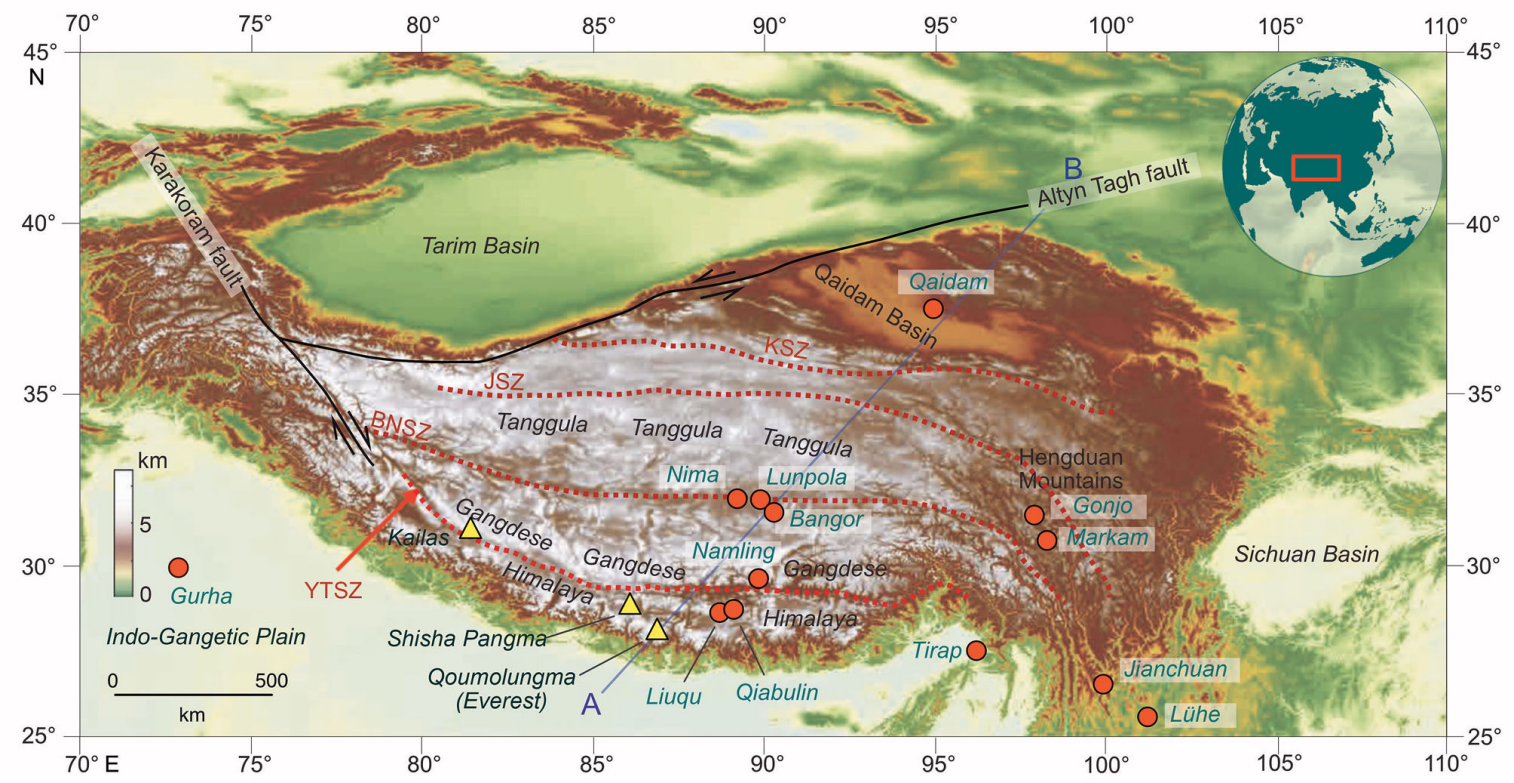
Map and inset showing the Tibet region including the main topographic features, faults bounding the Tibetan Plateau to the north and west and the main sutures: YTSZ, Yarlung–Tsangpo suture zone; BNSZ, Bangong–Nujiang suture zone; JSZ, Jinsha suture zone; KSZ, Kunlun suture zone. Plant fossil localities referred to in the text are shown as red circles. Yellow triangles denote the positions of mountain peaks referred to in the text.

Over an area of 2500 000 km^2^, the modern Qinghai–Tibetan Plateau (Fig. 1) is the most extensive elevated surface on Earth. Averaging in excess of 4500 m above mean sea level, the plateau extends 1000 km southward from the Altyn Tagh fault to the Yarlung–Tsangpo suture zone (YTSZ), south of which is the Himalayan thrust belt. Westward the plateau boundary is marked by the Karakoram strike-slip fault, while 2000 km to the east the plateau morphs into the Hengduan Mountains and ramps down into Yunnan and Sichuan. The presence of the plateau is thought to exert profound influences on the Asian monsoon systems, and by extension Asian biodiversity, so understanding the evolution of Tibetan topography is critical for exploring the links between them. Here, we review the topographic evolution of the Tibetan region as a complement to recent comprehensive reviews of the geology [1,2] because it is topography that exerts changes in atmospheric dynamics and provides a three-dimensional landscape within which the terrestrial biota functions and evolves.

The phrase ‘the uplift of the Tibetan Plateau’ permeates the scientific literature, extending even into the realms of molecular phylogeny [3]. It implies that this enormous and almost flat-surfaced portion of Earth's surface rose as a coherent entity, but this unlikely scenario has gained traction largely through simplistic modeling despite long-standing evidence that shows Tibet evolved in piecemeal manner [4–8]. Moreover, within this concept of a rising plateau it is not unusual to find Tibet and the Himalaya combined into a single entity, yet by conflating their separate geological histories the complex interactions between deep crustal processes, topography, climate and biodiversity [9] become obscured.

Linked to the simple monolithic uplift concept is the idea that Tibet rose purely due the collision of India with Eurasia (e.g. [10]), and that uplift occurred in the late Neogene (Miocene–Pliocene) [11–15] despite the onset of the India/Eurasia continental collision occurring in the Paleogene [10]. However, not all Tibet's topography was formed by the India–Eurasia collision and long before the arrival of India earlier terrane accretions must have produced significant uplands and so thickened crust existed across the Tibetan region. This review is aimed at a broad audience beyond the geological community and synthesizes a range of geological, isotopic and paleontological literature to better understand the topographic evolution of the Tibetan region, and hopefully put to rest some of the misconceptions that have become embedded in scientific literature across many disciplines.

To aid conceptual clarity, some explanations of terms are necessary. Here, we shall refer to the Himalaya–Tibet–Hengduan mountain area as the ‘Tibetan region’ because the terms relating to the modern topography, and particularly references to a ‘plateau’, are not appropriate before the present. Moreover, the terms ‘Tibetan Plateau’ and ‘Qinghai–Tibetan Plateau’ are reserved for the present day only, simply because it should not be assumed that an elevated expanse of low-relief topography existed before recent times (i.e. in this context, the last 10 million years). Instead, the term ‘Tibet’ relates to the area occupied by the modern plateau irrespective of its topography and does not imply any administrative or political boundaries. Lastly, it is important to understand that ‘uplift’ means movement along a vector opposite to that of gravity and involves work in terms of an increase in potential energy, while ‘exhumation’ and ‘unroofing’ do not involve such work and thus are not equated with uplift, nor do they necessarily imply a change in surface elevation [16]. The elevation of a landscape surface can also occur through sediment infilling of a basin and here again,

because no work is done against gravity, this process cannot be referred to as ‘uplift’. The significance of this distinction is important in the context of forming the modern low-relief Tibetan Plateau.

A notable feature of the modern plateau is its comparative lack of internal relief, or ‘flatness’ [6], and if the surface elevation of southern Tibet was not uniformly low early in plateau development it is unlikely that Tibetan uplift was a single event [17,18], yet because this concept is so pervasive it is useful to investigate briefly how it came about. A starting point for the synthesis presented here is the observation that ‘the geological history of the Himalaya and Tibet does not conform to monotonic models of intercontinental collision and plateau growth in time and space; instead, a wide variety of tectonic modes have operated over the past ∼220 Ma to produce this remarkable orographic feature’ ([1], p. 162).

### Models of the evolution of Tibet: simple crustal thickening

Early attempts to model the Tibetan orogeny widely assumed that the convergence between India and Eurasia would have been accommodated by crustal thickening (e.g. [19]), and that if the crustal thickness could be calculated surface uplift could then be estimated by applying the principle of isostacy. A significant unknown in this model, recognized at the time, was the extent of Indian crust (Greater India) that had been subducted. The shape and size of Greater India has been debated extensively [20] since the concept was proposed [21], with a recent estimate of post-collisional shortening being as much as 3700 ± 500 km [22].

This simple crustal shortening model inherently treats Tibet as a single entity that extends as far as the crust is thickened and does not allow for any lateral spreading of the crust or escape tectonics along the strike-slip fault systems of northern and eastern Tibet [23]. Moreover, it assumes that the lithosphere is not modified at depth by mantle processes [13,24].

### Models of the evolution of Tibet: lower lithosphere modification or ‘soft Tibet’

**Figure 2.**
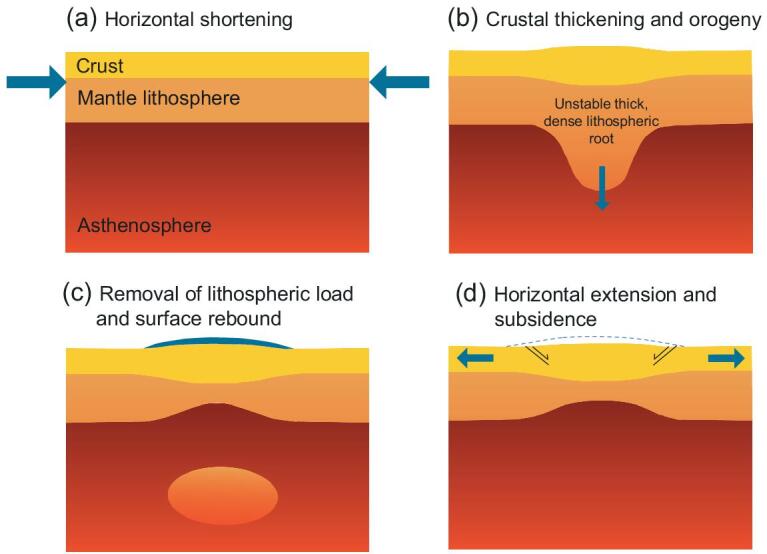
Cartoon showing the concept of mantle lithosphere thinning. (a) South–north shortening from the accretion of India thickens the Tibetan crust depressing the lower mantle lithosphere as in (b), which eventually falls away (thermally erodes) into the hot aesthenosphere as in (c) causing surface rebound (uplift). If not maintained, this elevated surface will suffer gravitational collapse as in (d) but, in the case of Tibet, is constrained by India's northward motion. Modified from [24].

Such a model of lower lithosphere modification was proposed by England and Houseman in 1988 [25]. Here, lithospheric thickening was envisaged as being accompanied by widespread viscous flow of both crust and mantle, and that after some degree of crustal thickening and isostatic compensation, mantle convection would trigger thinning at the base of the thickened lithosphere (Fig. 2). Loss of a cold (dense) lower lithospheric mantle (Fig. 2c) would result in rapid isostatic rebound, elevating the surface within a few million years (Fig. 2d) [25,26]. This process of convective thinning was envisaged to have produced significant uplift (e.g. from 3 to >5 km) over a large enough area to intensify the monsoon at ∼10–8 Ma [27].

This model predicts that Tibet rose as a single entity and that this took place no earlier than 10 Ma, i.e. in the late Miocene. However, the radiometrically dated middle Miocene (15 Ma) fossil leaf flora of the Namling–Oiyug Basin (Fig. 1) evidences a cool temperate paleovegetation, which, at the relatively low (tropical) latitude southern Tibet occupied throughout the Neogene, means it grew high above sea level (∼5 km) [28].

Quantitative paleoaltimetry, first using this well-dated flora [29] and subsequently using stable isotopes [30], showed the elevation of the Namling Basin at 15 Ma was indistinguishable from that of the present, so if uplift through lower lithosphere modification did occur it must have been before then, and so not connected to the perceived Miocene intensification of the monsoon.

### Models of the evolution of Tibet: the stepwise development of Tibet

**Figure 3.**
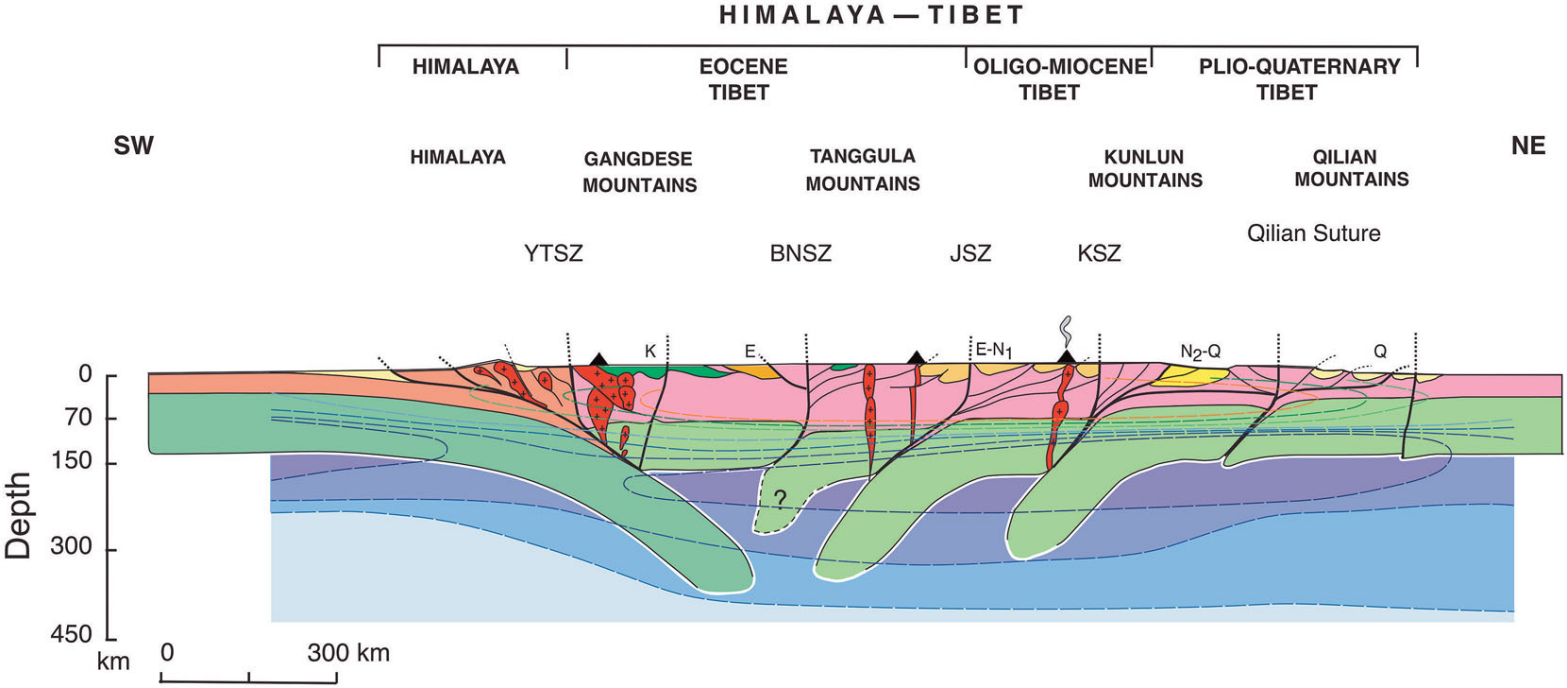
Cartoon cross-section of Tibet simplified from [7] invoking a stepwise formation of the Tibetan region. Although this model does not discuss explicitly the timing of the rise of the Himalaya, it does propose uplands developing first in southern Tibet and then progressing northward influenced by pre-existing deep structures and ‘bathtub sedimentation’, in which enclosed valleys fill with the erosion products of the surrounding uplands and reduce relief.

Convective lower lithosphere modification is not the only mechanism proposed for building Tibet. In 2001, Tapponnier *et al.* [7] expressed concern that the previous model, which they called a ‘soft Tibet’ model, ignored a number of large deep-rooted strike-slip faults that must have had a significant role in controlling the growth of an elevated Tibet. They argued that these faults suggest ‘a form of hidden plate tectonics’ (p. 1676), and a more brittle Tibet. They envisaged an oblique, three-phase, stepwise growth of Tibet from the southwest progressively toward the northeast, with India's relentless northward passage re-invigorating pre-existing suture zones as ‘mantle megathrusts’ (Fig. 3). This was recognition that Tibet's complex past would be important in determining how it would react under compression from India [31]. Another important component of their model was the extrusion eastward of a zone of pre-existing high relief near the northern edge of the Tanggula Mountains. This process, they argued, would have led to the development of the Red River and Xianshuihe shear zones.

**Figure 4.**
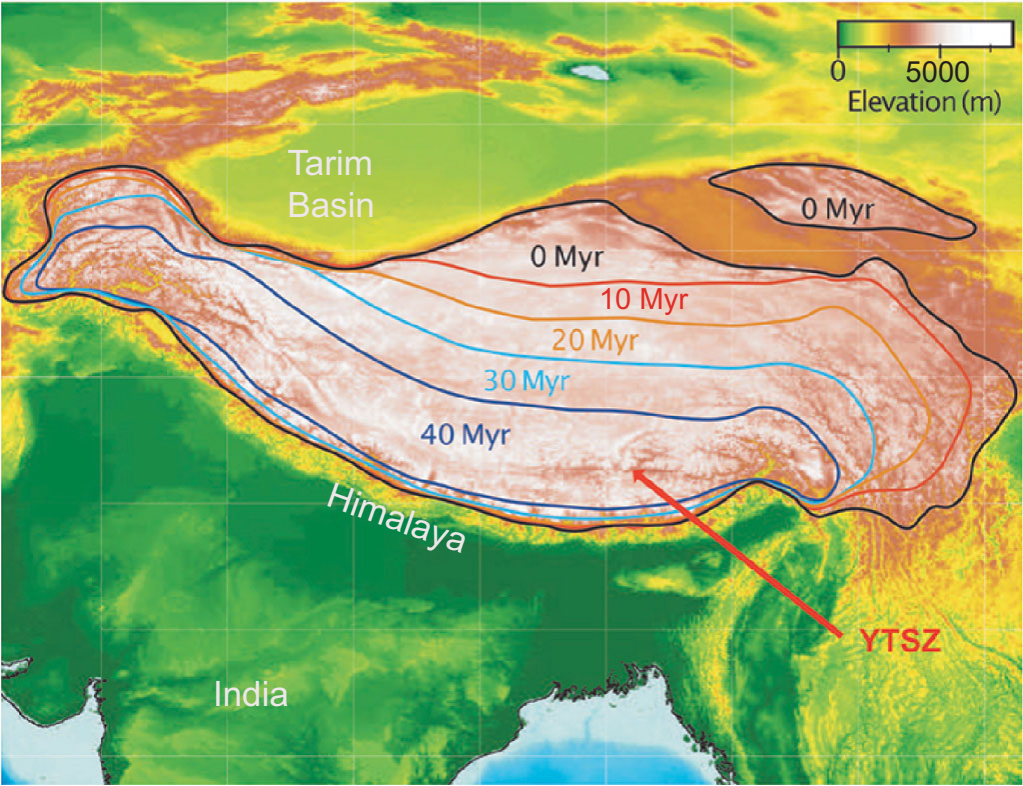
Progressive uplift of Tibet as illustrated in a commentary [32] on a review of stable isotope paleoaltimetry [33]. Note that as in Fig. 3 there is progressive uplift from north to south, but pre-existing structures are largely ignored such that the area uplifted by 40 Ma includes both large parts of southern Tibet and the Himalaya, despite the YTSZ separating the Eurasian plate from the Indian plate.

Similar to the stepwise model [7] is the concept of uplift beginning in the southwest of the region and progressing north-eastward: a pattern that seemingly emerges from stable isotope paleoaltimetry [32,33]. Here, an area encompassing much of the modern Himalaya, the YTSZ and much of southern Tibet was envisaged to have risen to above 4 km by 40 Ma, then another zone to the northeast to have achieved 4 km at around 30 Ma and so on to the northeast, where only recent uplift was invoked (Fig. 4). However, this model, like the soft Tibet model before it, largely ignores the pre-existing deep-rooted structures within Tibet: boundaries of the uplifted regions do not coincide with major suture zones and seemingly reflect supposed progressive crustal thickening as if Tibet behaved as a coherent entity.

### Models of the evolution of Tibet: the concept of a ‘proto-Tibetan Plateau’

**Figure 5.**
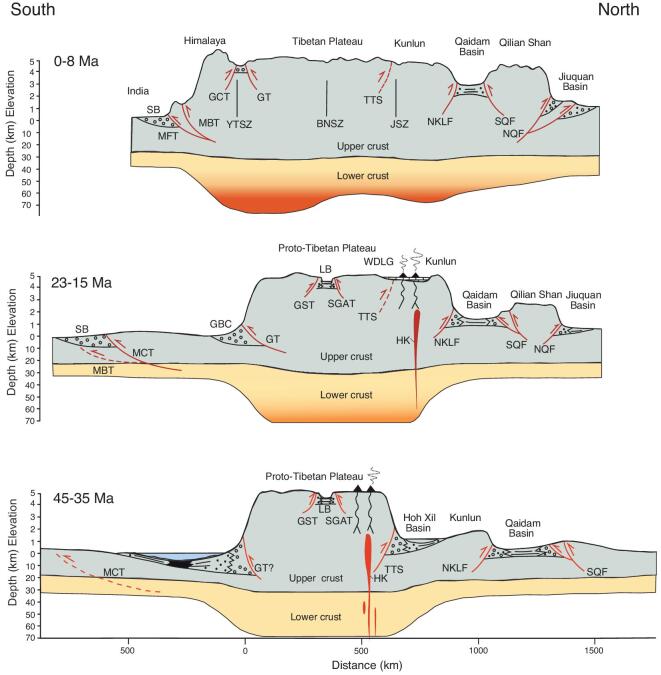
Progressive outward growth model of Wang *et al.* [10]. In this model, a central core of Tibet was elevated early in the Paleogene, while the Qilian Shan in the north and the Himalaya in the south are very recent constructs. Abbreviations: MBT, Main Boundary Thrust; GCT, Great Counter Thrust; TTS, Tanggula thrust system; NKLF, North Kunlun Fault; NQF, North Qilian Mountain Fault; GBC, Gangrinboche conglomerates; YTSZ, Yarlung–Tsangpo suture zone; BNS, Bangong–Nujiang suture zone; JS, Jinsha suture zone; MCT, Main Central Thrust; SL, sea level; GT, Gangdese Thrust; GST, Gaize–Siling Tso Thrust; LB, Lunpola Basin; SGAT, Shiquanhe–Gaize–Amdo Thrust; SQF, the South Qilian Mountain Fault; SB, Siwalik foreland basin; MFT, Main Frontal Thrust; HK, high-*K* calc-alkaline volcanics; AR, adakite volcanics; WDLG, continental Wudaoliang Group.

The seeds of the idea of an outward growth of a ‘proto-Tibetan Plateau’ were sown in the commentary by Mulch and Chamberlain in 2006 [32], but they did not use the term and they included the Himalaya in the area supposedly uplifted first (Fig. 4). Based on an extensive review of the literature, Wang *et al.* [10] proposed more comprehensively that Tibet developed by outward growth, both to the north and to the south, from a central elevated Paleogene ‘Proto-Plateau’ (Fig. 5). They argue that the central core of Tibet was already high (≥4.5 km) by 40 Ma (Eocene), and that further uplift subsequently occurred both to the north and to the south, including the development of the Himalaya after 15 Ma, as well as eastward growth as in the Tapponnier *et al.* [7] model.

The evidence for a high core of Tibet is multi-stranded. Numerous authors agree that the southern edge of Tibet featured an Andean-type Gangdese mountain range before the India–Asia collision (e.g. [4,34–37]) and that thermochronology suggests a slow cooling and erosional exhumation in central Tibet since the Eocene [38]. Added to that are the outcomes of isotope paleoaltimetry, which all indicate ‘high and dry’ (≥4.5 km) ‘altiplano’ Paleogene surface elevations [33,39]. The lack of crustal shortening in the late Cenozoic, low precipitation, cool temperatures and minimal erosion, as well as lithospheric processes, were all invoked to explain the flatness of central Tibet [10]. The concept of a high ‘proto-Tibetan Plateau’ covering up to two-thirds of the current area of Tibet as early as the end of the Cretaceous has been suggested [2], but this is contradicted by paleontological finds [40].

## BACK TO BASICS: THE ASSEMBLY OF TIBET

**Figure 6.**
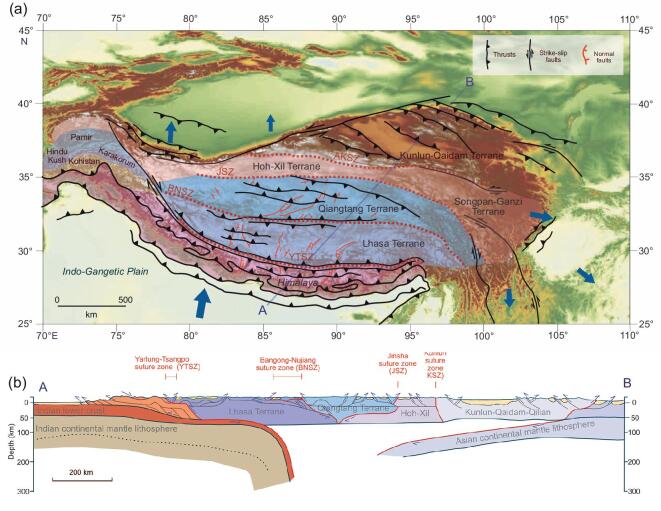
(a) Map of the Tibetan region showing the principal accreted terranes, thrusts, strike-slip and normal faults. The sizes of the blue arrows represent the relative motion today as measured by GPS [10]. (b) Modern topographic profile along the transect A–B in (a). (c) Hypothesized cross-section along the transect A–B in (a) simplified from [1].

It has long been known that Tibet is not a single monolithic block but an amalgam of Gondwanan terranes successively accreted onto the Eurasia plate. This assembly began in the early Mesozoic and continued to the early Cenozoic [2,5,18,31,34,35,41], India being the most recent of these terranes to arrive. From north to south across what are now the Tibetan Plateau and the Himalaya, the major accreted terranes are the Kunlun–Qaidam, Hoh Xil–Songpan Ganzi, Qiangtang, Lhasa and India (Fig. 6). Between these blocks, again going from north to south, are major suture zones: the Ayimaqin–Kunlun suture zone between the Kunlun–Qaidam and the Hoh Xil–Songpan Ganzi terranes, the Jinsha suture zone between the Hoh Xil–Songpan Ganzi and the Qiangtang terranes, the Bangong–Nujiang suture zone (BNSZ) between the Qiangtang and Lhasa blocks, and the YTSZ between the Lhasa block and the Himalayan thrust belt (Fig. 6).

**Figure 7.**
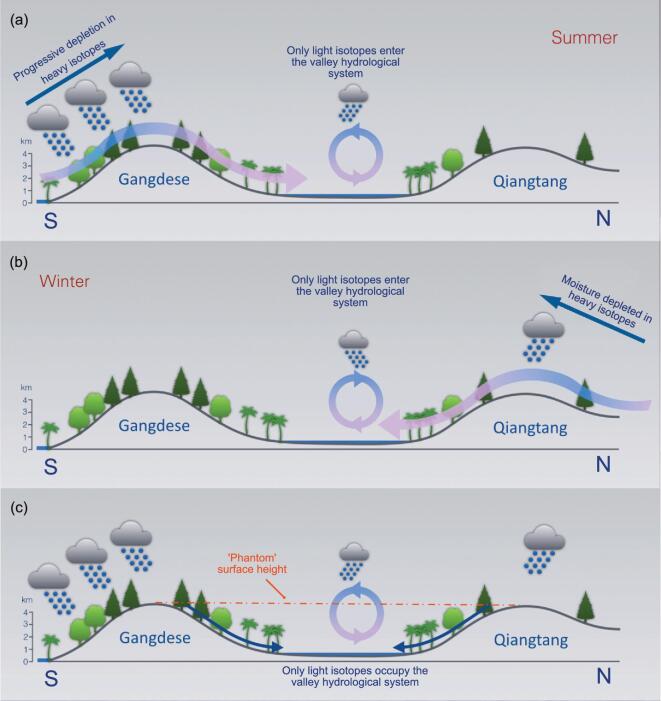
Cartoon illustrating the origin of ‘phantom’ elevated surfaces in stable isotope paleoaltimetry. (a) Summer winds from the south deposit heavy isotopes as the moist air parcel rises against the windward slopes of a mountain range, in which case the Gangdese before the rise of the Himalaya, so that only isotopically light moisture crests the range to enter the leeward valley. (b) Similarly, in winter heavy isotope depletion takes place on the windward side of the Qiangtang (Tanggula) Mountains, and again only isotopically light water enters the valley. (c) Isotopically light minerals and organic matter preserved in the valley system therefore seem to suggest a ‘phantom’ elevated surface that reflects the height of the bounding mountain ridges.

The onset of these collisions has been dated to the late Triassic to early Jurassic for the Kunlun–Qaidam and Qiangtang terranes [42,43], the early Cretaceous for the Qiangtang and Lhasa collision [44] and the early Paleogene for the initiation of the India–Lhasa block collision, which is ongoing (see [10] and references therein). However, there are significant outliers in the timing of the initial India–Lhasa terrane collision spanning 65 Ma [41,45] to 20 Ma [46].

The timing of the onset of continental collision and establishment of a land bridge facilitating terrestrial biotic exchange between India and Eurasia has long been a topic of contention and debate [1,2,10] and knowing when this event occurred is important because, apart from the biotic consequences, contact curtails pre-existing oceanic circulation patterns, sea temperatures and sea surface isotopic compositions. A recent molecular phylogenetic study using dated fossils to constrain an analysis of 50 mammalian lineages argues for free exchange between India and Eurasia as early as 64.8–61.3 Ma and thus in the Danian [47]. This is at the oldest extreme of the likely continental collisional age (55 ± 10 Ma) that emerged from the review of Wang *et al.* [10], but it does not imply land connection and loss of marine deposition along the whole length of the southern margin of Tibet, because the most recent marine sediments in the central part of southern Tibet only disappear at ∼55–50 Ma [45].

As with the India–Lhasa block collision, each of the previous accretion events would have thickened the crust, subducted ocean and continental lithosphere, and resulted in some increase in surface relief, so when India arrived Tibet already exhibited an inherited range of surface heights and complex underlying geology. Unsurprisingly the terrane amalgam shot-through by deep-rooted suture zones and faults was never likely to produce a Tibet that behaved coherently under compression [7,48]. Guillot *et al.* [2] estimate that by 50 Ma across roughly two-thirds of the plateau crust had already thickened to ∼50–55 km with isostacy producing average surface elevations of 2500–3000 m, and locally elevations exceeding 4000 m. They propose that after 50 Ma the northward ∼1000 km convergence of India resulted in a shortening of the Tibetan terrane amalgam by ∼40%, a thickening of the crust to ∼70 km and a further rise in surface elevation to the present mean of ∼4800 m.

While the crust on average may have been thus thickened and, on average, isostatic compensation may result in such mean elevations, the process of shortening produced by India's northward motion will have produced different responses in different parts of the Tibetan region at different times in an idiosyncratic manner [48]. A combination of fault/suture re-activation, local subduction, slab break-off, lower lithospheric ductile flow and even thermal delamination would have produced a complex surface relief and also accounts for oceanic slabs beneath Tibet [49] not necessarily associated with the collision of India.

Recent improvements in our understanding of regional geology, fossil biotas and, crucially, radiometric dating have transformed our understanding of Tibetan orography and a new pattern of topographic evolution across the Tibetan region is emerging.

## PALEOGENE TOPOGRAPHY OF TIBET

At the start of the Paleogene, Tibet exhibited significant topography relief as a result of prior accretions of the Songpan Ganzi–Hoh Xil, Qiangtang and Lhasa terranes [1,2]. The southern margin of the Lhasa terrane was somewhat further south than at present, perhaps even as low as ∼10°N [50–52], although data allowing for compaction-induced latitudinal shallowing put the Xigatse fore-arc basin, on the southern Lhasa terrane [1], at 16.5 ± 4°N in the early Cretaceous [53]. For the Lhasa terrane to have drifted southward since then is unlikely considering India's northward passage.

### The rise of the Gangdese Mountains

The Gangdese mountain system long predated the Lhasa–Qiangtang terrane collision, where suturing was diachronous from east to west and closure occurred in the Nima region by 118 Ma [1]. At that time, the Gangdese already formed a ‘Cordilleran-style orogen’ [1,4,54–57], but the elevation is not yet quantified. In Aptian–Albian times (125–100.5 Ma), the north central Lhasa terrain was below sea level as evidenced by marine platform sediments of the Takena Formation [44,58,59]. By ∼92 Ma, marine deposition ceased and this area emerged from the ocean as evidenced by the deposition of the unconformably overlying Campanian (∼83–78 Ma) lacustrine–fluvial, slightly evaporative, Shexing red beds with stacked paleosol horizons (referred to as the Lhunzhub member by Leier *et al.* [58]). The Shexing sediments were mainly derived from the south, i.e. the rising Gangdese [58]. This marine to non-marine transition is not just a single basin-scale phenomenon but is also recorded some 300 km to the west in the Coqen Basin when it took place at ∼96 Ma [15]. These transitions, coupled with the onset of development of the Xigatse fore-arc basin at 113–110 Ma [60], suggest that the Gangdese arc exhibited a significant rise in mid-Cretaceous time, but there are no quantitative estimates of its crest height.

By the Eocene, continued northward subduction of the Neotethyan oceanic lithosphere formed an east–west trending ‘Andean’-type Gangdese range that had reached an elevation of ∼4.5 ± 0.4 km by 56 Ma [37]. This confidence in a high topography arises from oxygen isotope analyses conducted on well-dated diagenetically unaltered paleosols, lacustrine calcareous carbonates and marls from the Linzizong Group, in the Linzhou Basin [37]. In the Eocene, the Gangdese Mountains were the first obstacle for moist air drawn northward in summer by the Siberian low: a seasonal depression that exists by virtue of Eurasia's position and size and the thermal capacity of land versus sea. On the windward side of the Gangdese, a Rayleigh isotope fractionation process would have operated, and because this process is predicable the height estimates are likely to be reliable [61].

This Gangdese highland did not extend northward to occupy the whole of the Lhasa terrane but was confined to its southern margin. In the northern part of the Lhasa block, rapid rock uplift must have been taking place between 80 and 70 Ma [62], but evidently was matched by erosion because there is evidence of large-scale bedrock peneplain formation between 70 and 50 Ma, and this surface has survived to the present [62]. Based on (U–Th)/He ages of apatite and zircon, and apatite fission track data, cooling and exhumation of Jurassic metasediments and Cretaceous granitoids took place between ∼70 and ∼55 Ma, with the exhumation rate falling rapidly from ∼300 to ∼10 m/m.y. between ∼55 and ∼48 Ma, after which erosion seems to have stabilized at a low rate, allowing the ancient surface (peneplain) to be preserved and rise as the rate of rock uplift exceeded the erosion rate. The planation process by laterally migrating rivers appears, initially, to have eroded 3–6 km of rock, suggesting the erosional surface remained at low elevation until the erosion rate reduced. This late Cretaceous rock uplift, and subsequent surface uplift, was presumably in response to crustal thickening produced by the India collision [62].

### Central Tibet in the Paleogene

In this work, ‘central Tibet’ is the region that lies between the Gangdese and Qiangtang uplands and centered along the BNSZ. Throughout the late Cretaceous and early Paleogene, the Gangdese mountain system likely stretched along most of, if not all, the full east–west extent of the Lhasa terrane along its southern flank. The northern Lhasa terrane was evidently at a lower elevation and was being subducted below the Qiangtang terrane along the BNSZ [1]. Determining how low the surface was either side of the BNSZ has turned out to be a vexed question, although the BNSZ and northern Lhasa terrane was uplifted above sea level by the development of the southward-younging Lhasa thrust belt in the mid-Cretaceous (∼95 Ma) [1,38].

The proto-Tibetan Plateau model [10] (Fig. 5) argues that the Gangdese, central Tibet and an elevated Qiangtang terrane formed the core of a central Tibetan highland, buoyed up by isostacy, that in due course expanded both northward and southward. All the other models infer some more uniform isostatic elevation in accordance with whatever crustal thickening/lithospheric thinning and other subsurface processes are envisaged. To test these models, it is essential to measure the surface height changes over time, especially in central Tibet.

### Isotope paleoaltimetry

Paleoaltimetry for central Tibet has mostly relied on stable isotopic compositions of rainfall and their relationships with elevation [63], but these are subject to significant uncertainty away from the windward slope of the southern flank of Tibet [61,64] and until recently this approach lacked the rigor imposed by climate model mediation. Stable isotope paleoaltimetry for central Tibet suggests elevations similar to those of present (>4 km) by 35 Ma (late Eocene) [33]. For example, micritic calcium carbonate paleosol nodules from the upper Niubao Formation in the Lunpola Basin (Fig. 1) along the BNSZ yield late Eocene elevations of $4850_{ - 460}^{ + 380}\,\,{\rm{m}}$, while slightly older limestones and marls within the same formation yielded an elevation of $4050_{ - 620}^{ + 510}\,\,{\rm{m}}$ [33]. Mid-Miocene thin-bedded lacustrine marls and limestones of the overlying Dingqing Formation, still within the Lunpola Basin, also gave high elevations of $4260_{ - 575}^{ + 475}\,\,{\rm{m}}$ [33]. In the nearby Nima Basin (Fig. 1), radiometrically dated soil carbonates from the Nima Redbed unit yielded a paleoelevation estimate of 4.5–5 km at 26 Ma (late Oligocene) [39]. How this proposed high-elevation plateau is supposed to have remained stable for so long has not been fully explained.

Other isotope systems have yielded similar results. Deuterium/hydrogen (D/H) ratios in *n*-alkanes of leaf waxes suggest paleoelevations of 3600–4100 m for the Niubao Formation and 4500–4900 m for the Dingqing Formation [65], very similar to those of oxygen isotopes in carbonates and ‘strongly supports the presence of similar precipitation isotopic compositions in both archives despite different isotope systems, source water reservoirs, archive materials, modes of incorporation, and diagenetic processes.’ ([65], p. 64). Subsequent review and re-examination of these results did not substantially alter the conclusions that Tibet was high in the Eocene, but neither was there evidence for a progressive northward elevation change [66].

Contrasting with these high elevations, which are virtually indistinguishable from those of the present, are the fossil finds from the BNSZ basins. The Lunpola Basin (Fig. 1) is a rich source of faunal (insect, fish, and mammal), floral (megafossil and pollen/spores) and molecular (biomarker) fossils. The Cenozoic sediments within the basin are some 4 km thick and comprise paleosols, fluvial, fluvio-deltaic and lacustrine units, some indicative of freshwater and some saline conditions [67]. The Cenozoic succession is divisible into a predominantly fluvial Paleocene–Eocene Niubao Formation and an overlying predominantly lacustrine Oligocene–Miocene Dingqing Formation [67,68]. Radiometrically constrained [69] magnetostratigraphy, cyclostratigraphy [68] and palynology [14] provide the chronology for the Dingqing Formation, but the surface geology is often complex as beds are folded and faulted [70].

Among the fossil finds from the Chattian (late Oligocene) lower Dingqing Formation is a climbing perch, *Eoanabas thibetana* (Anabantidea) [71], whose modern relatives occupy tropical lowlands of South Asia and sub-Saharan Africa below 1000 m, while higher in the succession (early Miocene–Aquitanian) a primitive form of the cyprinid fish *Plesioschizothorax macrocephalus* has been recovered, whose modern relatives are restricted to elevations below 2500 m. These and other discoveries [40] suggest low elevations in marked contrast to the >4000 m derived from isotope studies.

### Palynology

The palynology of the Dingqing Formation [14] spanning 25.5–19.8 Ma comprises throughout a mixture of cool temperate and more thermophilous taxa that are usually found in subtropical environments. Such a taxonomic mix does not reflect co-occurrence in the vegetation but mixing of palynomorphs during transport into the lake sediments. The paleoelevation obtained from the total palynological assemblages using an estimated Eocene free air lapse rate was 3190 ± 100 m [14]. However, this reflects not the elevation of the lake margins but the blended heights of the source vegetation communities, including montane taxa, and thus represents the height of an undefined location between the basin lake and the crests of the surrounding mountains [70] even though the estimated height is lower than that given by isotopes. By virtue of the long distances pollen and spores can travel without showing any signs that such pre-depositional transport has taken place, pollen/spore assemblages inevitably represent a mixture of taxa growing in different environments and in particular different elevations. As a consequence, they are unreliable proxies for quantitative paleoaltimetry, unlike larger and more delicate plant parts such as leaves, which readily show signs of transport prior to burial and thus better reflect vegetation nearby the site of fossilization.

### Plant megafossils

Plant megafossils from near the base of the Dingqing Formation of the Lunpola Basin (Fig. 1) represent typical subtropical to warm temperate taxa favoring humid conditions. These include a palmate palm leaf, *Koelreuteria lunpolaensis*, *K. miointegrofolia* (golden rain tree), a *Pistacia* leaflet, *Ailanthus maximus* fruits, a whole plant of *Limnobiophyllum pedunculatum* [72], a winged fruit of *Cedrelospermum*, a palmately compound leaf of *Handeliodendron* sp., several unidentified toothed and untoothed woody dicot leaves and the lake margin monocot *Typha* [71]. The flora as a whole consists of intact leaves and leaflet clusters and shows no sign of long-distance transport, so represents vegetation growing spatially and altitudinally very close to the large margin [73].

Because palms are intrinsically cold-intolerant, they can indicate a maximum possible elevation for the basin floor provided that the cold month mean terrestrial thermal lapse rate is known [70]. Large (∼1 m long) fronds of the coryphoid palm *Sabalites tibetensis* were recovered from lake sediments of the Dingqing Formation estimated to date from ∼25 Ma [70]. Using 13 possible topographies for central Tibet, climate modeling with Chattian boundary conditions showed that the only landscapes compatible with palm winter survivability were those with deep central valleys with a valley floor height <2.3 km above sea level [70]. This model-mediated approach avoids the use of inappropriate free air lapse rates and automatically compensates for secular climate change, in terms of both temperatures at a sea level datum and thermal terrestrial lapse rates.

While the lowermost (Paleocene) units of the Niubao Formation host aeolian sands and other indicators of aridity, the middle Eocene (Lutetian) part has recently yielded a diversity of plant remains including leaves, fruits and seeds [74,75] from lacustrine units within an otherwise fluvially dominated succession in the Bangor Basin (Fig. 1). These remains point to a humid subtropical (and therefore low elevation) flora with floristic links to the Eocene Green River flora, western USA, and the middle Eocene Messel flora, Germany [76]. One additional piece of evidence pointing to lowland thermophilic humid forests in the Paleogene of central Tibet includes finds of amber from dipterocarps (tropical lowland forest dominants across South and Southeast Asia today) that might have been reworked from the Niubao into the Dingqing Formation [77], but so far no definitive dipterocarp megafossils have been found.

### The Qiangtang uplands

To the north of the BNSZ were the Qiangtang (Tanggula) uplands. Quantifying the elevation history of these highlands is more challenging than for the Gangdese because of their continental interior position, but they have been estimated to have reached an elevation of ∼5000 m by 28 Ma [78]. The continental effect on the isotopic composition of rainfall is complex in this inland setting and can only be properly resolved using appropriately configured isotope-enabled climate models. Furthermore, it is not yet possible to say when this elevation was achieved, but the uplands were shedding sediment northward in the Paleogene as evidenced by deposits in the Hoh Xil Basin [8,79] and suggest that the Qiangtang terrane may have supported an east–west mountain chain throughout the Eocene. However, the surface height of this upland needs to be re-examined using appropriately configured isotope-enabled models that allow not only time-specific isotopic values of source waters but also trajectories of source moisture to be more accurately determined, something that was not available when the original elevation estimate [78] was made.

### Reconciling isotopic and paleontological paleoaltimetry

There have been few instances of multiproxy cross-calibration, but one location that has been studied intensively is the mid-Miocene (15 Ma) Namling–Oiyug Basin (Fig. 1) within the Gangdese highland, south central Tibet. Here, radiometrically dated leaf fossils and lacustrine/paleosol carbonates have both been used for paleoaltimetry. The first investigation derived paleo-moist enthalpy from leaf form (physiognomy) using CLAMP (Climate Leaf Analysis Multivariate Program, http://clamp.ibcas.ac.cn) analysis from what has become known as the Namling flora: a cool temperate, predominantly deciduous lacustrine leaf assemblage from a single inclined horizon spanning modern elevation of 4300–4700 m [28,29]. Lacking a proximal contemporaneous sea level flora, a climate model was used to provide the required sea level datum from which a paleoelevation of 4689 ± 895 m was obtained [29]. Subsequently, this was revised to 5260–5540 m using a calibration more suited to potentially monsoonal climates and a near sea level datum from a similar-aged flora in the Siwaliks, northeast India, corrected for paleolatitude [80]. This value is remarkably similar to an elevation of $5200_{ - 606}^{ + 1370}\,\,{\rm{m}}$ obtained using the Rayleigh fractional model on oxygen isotopes within carbonates at the same location [30], as well as a value of $5100_{ - 1900}^{ + 1300}\,\,{\rm{m}}$ subsequently obtained from hydrogen isotopes in leaf waxes [81]. All these measurements, both paleontological and isotopic, are identical within methodological uncertainties, so why do isotope and paleontological proxies give such divergent results in central Tibet?

**Figure 8.**
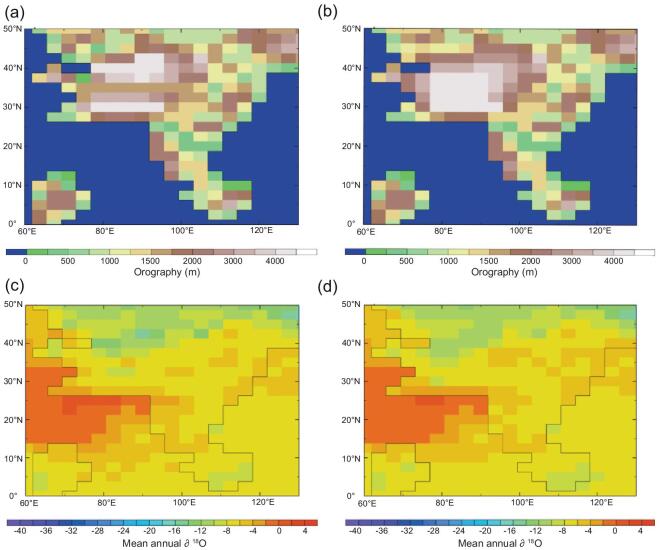
Maps of southern Asia (India is in the lower left corner) showing the results of an isotope-enabled climate model simulation for the Lutetian (middle Eocene). (a) Orography with a deep valley system located at ∼35°N. (b) A simple high plateau set at 4.5 km. (c) ∂^18^O distribution map for (a). (d) ∂^18^O distribution map for (b). There is no valley signature in (c), which appears very similar to the high-plateau scenario (d) showing that the isotopes entering such a valley reflect the height of the bounding mountain systems and the lowland between them appears as a plateau.

To answer this question, we begin from the standpoint that the paleontological data indicative of humid subtropical conditions are incompatible with a ‘high and dry’ scenario for central Tibet in the Paleogene. Instead, the fossils evidence a diverse lowland ecosystem. We envisage a predominant Indian Ocean moisture source to the south and a summer northward air parcel trajectory driven by an Asian interior (Siberian) low-pressure system (Fig. 7a). Here, moisture-laden winds would encounter the east–west Gangdese mountain system and be forced upward preferentially raining out the heavy isotopes, leaving light isotope enriched moisture to crest the mountain tops and enter the lowland to the north. Similarly, in winter cool dry air from the north, passing over land and the Qiangtang mountains, will have depleted heavy isotope content (Fig. 7b). This means that the lake and soil carbonates of the lowland between the Gangdese and Qiangtang ranges will be isotopically light and therefore yield a ‘phantom’ high elevation reflecting that of the mountain ridges surrounding this great central valley (Fig. 7c). This effect is shown by isotope-enabled computer models (Fig. 8) even at a coarse spatial resolution. Because prior to the rise of the Himalaya the Gangdese formed a southern highland of a similar height to today's plateau, and conceivably hosted peaks approaching the heights of many modern Himalayan peaks, the sediments preserve isotopic ratios indicative of paleoelevations similar to those of today.

### Northern Tibet

Despite evidence that crustal shortening began more or less synchronously across the Tibetan region in the Eocene [41], in all but the ‘soft Tibet’ model [7] (Fig. 2), northern Tibet is supposed to have been the last area to rise. Stable isotope paleoaltimetry indicates the Qaidam Basin (Fig. 1), now at an elevation of between 2.8 and 3.2 km, was low in the Paleogene and only rose in the Miocene [82]*.* However, there is some evidence that uplift in parts of northern Tibet occurred much earlier, e.g. the appearance of high-altitude derived pollen in the Xining Basin [83] and a positive shift in oxygen isotope values in the Qaidam and Tarim basins before 38 Ma [82,84]. The recent discovery in the northern Qaidam Basin of a cool temperate predominantly deciduous early Oligocene flora also challenges that view [85], as does hydrogen isotope data from the Hoh Xil Basin [86], although like other inland stable isotope paleoaltimetry this needs to be re-evaluated using isotope-enabled climate model mediation to better quantify the source of isotopic composition and the air parcel trajectory/continental effect [61]. This implies an Eocene uplift of the region or a pre-existing uplift derived from pre-Cenozoic terrane collisions. An Eocene rise is more likely as it is consistent with thermochronological studies indicating Eocene exhumation/cooling in the nearby Beishan Mountains [87–89] and more wide-scale deformation across Tibet at 52–48 Ma [90]. Additionally, other areas of northern Tibet also seem to have risen in the Paleogene [83]. Taken together, this implies significant deformation and uplift in parts of northern Tibet during the Eocene, seemingly simultaneous with eastward extrusion of Tibet and the building of the Hengduan Mountains.

### The rise of eastern Tibet and the Hengduan Mountains

It has long been recognized that a significant proportion of north–south shortening under compression from the India–Asia collision may have been accommodated by extrusion of parts of Tibet to the east and southeast [23,91–93]. This extrusion was originally envisaged to have taken place in a somewhat rigid manner but the low-relief, high-elevation topography in eastern Tibet has been used as evidence for ductile flow of the lower crust [94,95].

A slightly different model based on paleomagnetic analysis envisages extrusion, translation to the south and rotation of the Indo-China block. Tong *et al.* [52] suggest that since the late Eocene the Lhasa and Qiangtang terranes have not experienced enough crustal shortening to have provided sufficient crustal material for the southward extrusion of southeastern (SE) Tibet. Instead, they suggest displacement of the Indo-China block that was situated to the north of the Qiangtang terrane until ∼43 Ma. Most recently, based on a high-resolution paleomagnetic study and previous work, Li *et al.* [90] have argued that crustal shortening and a 30° clockwise rotation took place in eastern Tibet between ∼52 and 48 Ma, coincidental with deformation, shortening and fault reactivation across Tibet at this time, and a slowdown in the northward motion of India (see references in [90]). They also argue for a further 30° clockwise rotation taking place after 41 Ma.

Controversy surrounds not only the building of SE Tibet but also the timing of its rise. Recently, a multi-phased rapid uplift of SE Tibet, starting as early as the late Cretaceous [96–100], has been suggested based on low-temperature thermochronological studies, following on from earlier work that suggested a predominantly Miocene uplift of the region based on river incision measurements [95,101]. Rapid incision of major river systems as revealed by low-temperature thermochronology is estimated to have taken place between 15 and 10 Ma and used as evidence for lower crustal flow [102,103]. However, incision may not be associated with surface uplift but an intensification of monsoon rainfall [104]. Eocene to Miocene provenance shifts have been studied to determine changes in river drainages (e.g. [101,105–107]), but all depend on a reliable regional dating framework.

Such a dating framework is also essential for paleoaltimetric studies, and until recently the baseline dating reference for the region has been geological maps lacking absolute age constraints. Instead, dating, and thus uplift studies, relied heavily on biostratigraphy, with an inherent element of circular reasoning. Similar, seemingly modern, floral and faunal assemblages preserved within the numerous (>200 [108]) Cenozoic basins in Yunnan led to most of them being regarded as Miocene and inevitably Miocene reference frames were used for isotope paleoaltimetry and paleoclimate determinations (e.g. [109,110]).

The first clue that regional dating required revision came from a study of the Jianchuan Basin (Fig. 1) by Gourbet *et al.* [111] who found that the Jinsichang, Shuanghe and Jianchuan formations regarded as Oligocene to Pliocene are in fact all late Eocene and that previous paleoelevation estimates assuming a Miocene age needed to be revised from 2.6 km [109] to 1.2 ± 1.2 km. Re-analysis shows a paleoelevation somewhere between these two estimates [64], but awaits further investigation in conjunction with isotope-enabled climate modeling.

Following the Jianchuan Basin re-dating, a primary ash was discovered in the Lühe Basin [112] (Fig. 1) with a zircon U–Pb age of 33 ± 1 Ma, and some 20 million years older than a late Miocene age previously assigned based on the modern aspect of the fossil flora (leaves, seeds and pollen) [113]. These ages are also consistent with those of detrital zircons found in fluvial sands higher within a mine section [107]. These radiometric tie points suggest the Lühe Basin formed at ∼35 Ma and importantly constrain the onset of movement of the basin-bounding Chuxiong fault to ∼35 Ma and thus contemporaneous with initiation of the Ailao Shan–Red River fault system [23,114].

The Mankang (Markam) Basin (Fig. 1) hosts another fossil flora previously thought to be Miocene [115,116], but now known to span the Eocene–Oligocene (E–O) boundary [117]. There are numerous plant fossils from several horizons within the basin, but two within the Lawula Formation are of particular note because they record climate and elevation at the end of the Eocene. The lower assemblage, representing evergreen and deciduous mixed subtropical to warm temperate vegetation, is bounded by volcaniclastics that based on ^40^Ar/^39^Ar ages of 35.5 ± 0.3 and 34.61 ± 0.8 Ma below and above respectively date it to the latest Eocene. An overlying assemblage characterized by much smaller leaves and indicative of cooler temperate vegetation is similarly constrained to be between 34.7 ± 0.5 and 33.4 ± 0.5 Ma, placing it at or just above the E–O boundary (33.9 Ma). Using moist enthalpy derived from CLAMP analysis of sea level floras in India (Gurha, Tirap, Fig. 1) and those in the Markam Basin, the lower assemblage yielded an elevation of 2.9 ± 0.9 km and the upper assemblage 3.9 ± 0.9 km, the latter being indistinguishable from the modern elevation of 3910 m. It is difficult to determine whether the apparent 1 km elevation increase in at most 3.5 million years across the E–O is genuine or to some extent reflects secular climate change, but active tectonics is consistent with widespread crustal shortening and igneous activity within the Qiangtang terrane at that time [44,118].

Previous stable isotope paleoaltimetry in the Markam Basin assumed an early Miocene age [110], yet returned a paleoelevation of $3.8_{ - 1.6}^{ + 1.1}\,\,{\rm{km}}$. These uncertainties, spanning 2.7 km, are very large and the exact age of the material yielding this result is unknown, yet accurate dating is essential given the potential uplift taking place during the deposition of the basin fill. Furthermore, because Miocene isotope lapse rates and air trajectories were assumed, the estimate has to be regarded as unreliable despite its similarity to that obtained from the leaf fossils. In recognition of the problems posed by incorrect age assumptions, Hoke [64] noted that, irrespective of the dating issue but using an estimated Paleogene isotopic lapse rate, the differences in preserved oxygen isotopes along NNW–SSE transect across Yunnan to SE Tibet yield an elevation difference of 4.5 ± 1 km from north to south.

Tectonism and motion to the SE are ongoing in the Hengduan Mountains and Yunnan (Fig. 6) and, as many low-temperature thermochronological studies show, may have also been significant in the late Miocene associated with lower crustal flow [119], increased monsoon rainfall [104], base-level fall caused by fault-related river captures [109,110] or a combination thereof. However, if near-modern relief across SE Tibet and northwest (NW) Yunnan was achieved by the end of the Eocene, when did most of the regional uplift occur?

Paleomagnetic studies of the Gonjo and Ranmugou formations in the Gonjo Basin, ∼100 km north of Markam in SE Tibet (Fig. 1), suggest the area was deformed in the early Eocene (54–50 Ma), co-incidental with other major deformation across Tibet, significant rotation of eastern Tibet and a slowdown in India's northward motion [52,90]. This was followed by ∼1300 ± 410 km of crustal shortening in the northern Qiangtang terrane after 35.4 ± 2.4 Ma suggesting uplift in northern Tibet taking place from at least the early Eocene through to where some near current elevations were achieved [85,86] before the Neogene.

### The rise of the Himalaya

For many years, references to ‘the uplift of Tibet’ have automatically, if sometimes subconsciously, included the rise of the Himalaya with little distinction made between the development of the plateau area and that of the Himalayan system. This is exemplified in Fig. 4 where large parts of the Himalaya are included with the Lhasa terrane and inferred to have achieved elevations >4 km as early as 40 Ma. However, this completely ignores the existence of the Yarlung–Tsangpo suture that marks the junction of the Indian and Eurasian plates and the geodynamics associated with the collision process. It is therefore essential that, when considering the orogeny of the Tibetan region, the formation of the Himalaya is treated separately from the development of Tibet.

In the Tapponnier *et al.* model [7] (Fig. 3), the timing of the rise of the Himalaya is not defined, but the model of Wang *et al.* [10] (Fig. 5) suggests that the Himalaya were essentially a Neogene construct formed after the core ‘proto-Tibetan Plateau’ was established in the Paleogene. Support for this Neogene rise of the Himalaya is long-standing and one of the first attempts at quantitative paleoaltimetry in the region was conducted nearly 50 years ago with the discovery of *Quercus semecarpifolia* (*Quercus* sect. *Heterobalanus*) remains of supposed Pliocene age at a reported modern elevation of 5.6 km on the 8027 m high Himalayan peak known as Shisha Pangma (also known as Gosainthān) [11]. This seemed to indicate a very recent rise of the Himalaya, but unfortunately these remains were not found *in situ* and their precise origin and exact age remain unknown.

**Figure 9.**
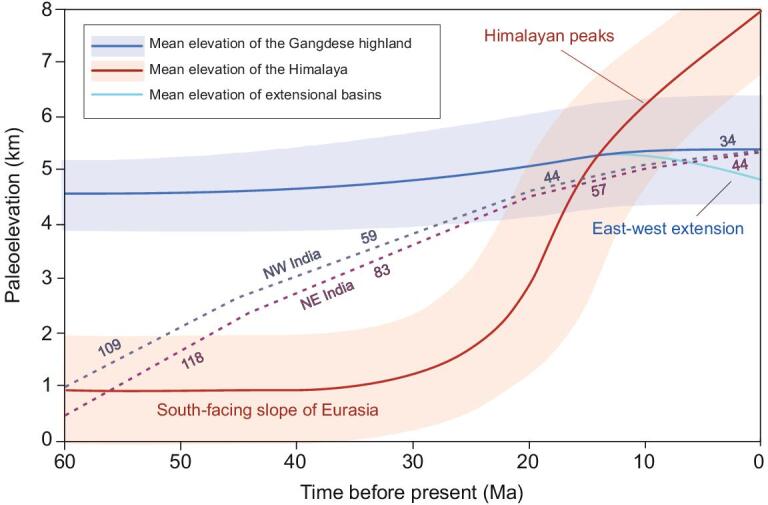
Diagram showing isotope and CLAMP paleoaltimetry for the southern flank of the Tibetan region. The red line is the inferred elevation of the Himalaya, while the blue line is the elevation of the Gangdese highlands. Shading is indicative of uncertainties. Modified from Xu *et al*. [122]. The rates of India's northward motion (dotted lines) are taken from Molnar and Stock [121].

As with other paleoaltimetric work in the Tibetan region, useful insights come from a combination of paleontological and stable isotope proxies. The growth of the Himalaya began in the early Eocene but it was not until ∼23 Ma that the locus of deformation caused by the India–Eurasia collision moved from Tibet to south of the Gangdese. This shift was accompanied by a rapid acceleration in Himalayan uplift [120] and a slowdown in India's northward motion [121] (Fig. 9). Ding *et al.* [120] and Xu *et al.* [122] used a combination of moist enthalpy derived from radiometrically dated plant fossils and oxygen isotope analysis to argue for the rise of the Himalaya against the southern flanks of the Gangdese (Fig. 9) beginning in the Eocene. Before ∼58.5 and ∼55 Ma, the area immediately to the south of the YTSZ must have been near sea level because the last marine units in the area date from that time [45], but soon thereafter Eocene (∼56 Ma) plants representing the tropical Liuqu flora [123] suggest an elevation of ∼1 km, rising to ∼2.3 km at 24–21 Ma based on the nearby warm temperate Qiabulin flora. A similar elevation was obtained from oxygen isotopes from the same deposits (2.5 km). Higher in the Qiabulin section (21–19 Ma), isotopes alone yielded an elevation of ∼4 km, because at that location there were no plant fossils. By ∼15 Ma, the Himalaya had risen to at least 5 km [124], matching, and perhaps beginning to exceed, the height of the Gangdese. Subsequently, continued growth of the central Himalaya has formed nine peaks in excess of 8 km with an average elevation across the range of ∼6 km. Thus, the influence of the Himalaya on atmospheric circulation (deflection of air parcel trajectories and a rain shadow effect over Tibet) only really began to operate from the middle Miocene onward [120]. Similar work along the length of the Himalaya is required to understand their lateral growth and their effect on climate and biotic systems.

### Summary

Recent fossil discoveries have shown that Tibet hosts a wealth of paleontological data attesting to a past where diverse Paleogene forests seemingly existed in subtropical intermontane lowlands in a great central valley along the Bangong–Nujiang suture zone between the Gangdese and Qiangtang uplands. These forests, with floristic links across the Northern Hemisphere, also provided a range of habitats for an abundant fauna. This suggests that only in the Neogene did a high plateau begin to form across Tibet by raising the valley floor to near the height of the bounding mountains through a combination of compressional uplift and sediment fill. The Tibetan Plateau was never uplifted as a monolithic entity but has evolved in a piecemeal fashion since early in the Mesozoic. Mesozoic terrane collisions formed a topographically complex landscape with deep valleys and high mountains providing a high ecological niche diversity that contributed to, and nurtured, modern Asian biodiversity.

**Figure 10.**
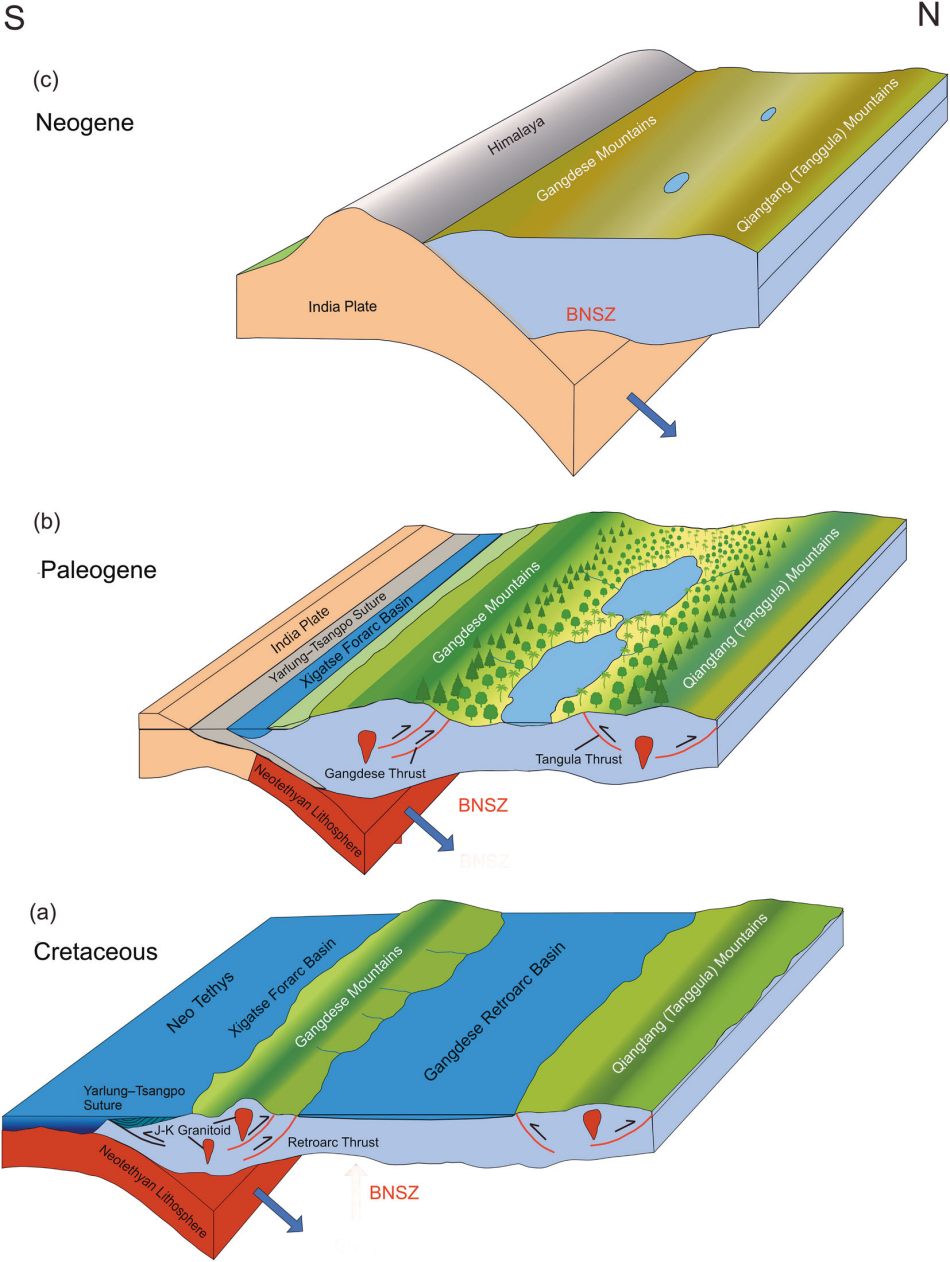
Cartoons illustrating the topographic development of central and southern Tibet as well as the Himalaya. (a) Southern Tibet before the arrival of continental India. (b) Middle Paleogene where a central Tibetan valley hosts large lake systems at elevations <2 km surrounded by a subtropical biota, while on the mountain flanks temperate woodlands give way to conifer-dominated cool temperate forests at elevations of 4 km or higher. (c) The near-modern (late Miocene) development of a high plateau in a cold dry climate formed by the rain shadow cast by the high Himalaya.

This progressive building of Tibet also thickened the crust, collision by collision, with the inevitable consequence that not all of that thickness, and elevation, can be attributed to the arrival of the Indian plate. This also means that the size of greater India was likely much smaller than often envisaged. When the Indian plate did arrive, Tibet already had a complex, and in places high, relief, the most dominant features being the east–west trending mountain ranges of the Gangdese and Tanggula (Qiangtang) uplands (Fig. 10a). After the sea retreated from the Gangdese Retroarc Basin in the middle Cretaceous, a wide semi-arid lowland separated the two upland regions forming a great central Tibetan valley with moisture from the south being blocked by the high (≥4.5 km) Gangdese Mountains. In the Eocene, more moisture entered the valley (Fig. 10b), most likely through increasing intensification of the South Asia Monsoon, and a humid monsoonal subtropical forest ecosystem developed that based on its composition likely existed at elevations below ∼2 km.

Tibet experienced significant north–south deformation during the Eocene, perhaps progressively halving the width of the central valley and causing localized uplift to near present heights in the north of Tibet, as well as uplift in the eastern Tanggula range. This compression was also translated eastward, forming the uplands of NW Yunnan that today comprise the Hengduan Mountains and host globally exceptional biodiversity. Major deformation in NW Yunnan during the late Eocene/early Oligocene initiated the major fault systems and formed much of the present relief, including many of the coal-bearing basins of the region. As resistance to further north–south contraction across Tibet increased, some deformation took place across the central region raising the valley floor to near-modern elevations by the middle Miocene, but by the start of the Neogene the main locus of deformation had shifted to south of the Gangdese where the Himalaya began to build rapidly, exceeding the ∼5 km height of the Gangdese, in the mid-Miocene (Fig. 10c), and they have continued to build and extend southward since then.

As the Himalaya passed through 5 km, they imposed an increasing rain shadow effect on central Tibet. By the late Oligocene, this caused the central valley lake margin vegetation to become more xerophytic, while Neogene uplift and ‘bathtub sedimentation’ [7] raised the valley floor to its present elevation of ∼5 km exacerbating the drying process.

All the phytopaleoaltimetry cited here, and some of the more recent isotope paleoaltimetry, has benefitted from various forms of climate model mediation. The pre-existing surface height estimates that did not use such an approach need to be re-examined with, and validated using, high-resolution coupled ocean–atmosphere–isotope–vegetation models before we can have a definitive understanding of the topographic development of Tibet. Knowing past topography is essential for disentangling the complex interactions between orography, climate and biodiversity, and future investigations of such interactions will require a move away from treating Tibet as a simple plateau rising as a block, but instead use paleotopographies that are as realistic as possible. Such work can be undertaken empirically and iteratively, but as model spatial resolution increases so does the requirement for accurate multiple quantitative paleoaltimetric proxies to be used in conjunction with each other in order to exploit their various different, but complementary, characteristics.

## CONCLUDING REMARKS

### Summary points

1. Tibet is not a monolithic entity but assembled piecemeal during the Mesozoic by successive terrane accretions.
2. This produced a complex high-relief landscape harboring subtropical biotas in deep valleys.
3. Stable isotope and paleontological paleoaltimeters measure different aspects of the topography: isotopes tend to reflect high elevations, while fossils tend to reflect lowland elevations.
4. In valley systems, isotopes seem to reflect the heights of the bounding mountain crests and the valley appears as a high plateau.
5. Contrary to previous conceptual models, Tibet did not rise as a pre-formed plateau, or by crustal thickening driven solely by the India–Eurasia collision, but evolved gradually through tectonic compression and internally drained basin sediment infill.
6. The Tibetan Plateau did not form until the Neogene, concurrently with the Himalaya rising above 5 km and the establishment of a strong rain shadow affect.

### Future issues

1. Future paleotopography will be best quantified using multiple altimetric proxies (stable isotope and paleontological) in combination, mediated by climate or Earth system models.
2. Existing stable isotope paleoaltimetry needs to be re-assessed using isotope-enabled climate models and empirical evaluation of past landscapes.
3. To fully understand the contribution the Tibetan region has made to Asian biodiversity and monsoon evolution requires further well-dated fossil collections in conjunction with Earth system modeling using realistic paleotopographies and not simple block-like representations of Tibet.

## FUNDING

This work was supported by the Second Tibetan Plateau Scientific Expedition and Research Program (STEP), Chinese Academy of Sciences (CAS) (2019QZKK0705), the National Natural Science Foundation of China (NSFC) (41922010 and 41872006), the Strategic Priority Research Program of CAS (XDA20070301, XDA20070203 and XDB26000000), the XTBG International Fellowship for Visiting Scientists to R.A.S., the NSFC–NERC (Natural Environment Research Council of the United Kingdom) Joint Research Program (41661134049 and NE/P013805/1) and the CAS 135 Program (2017XTBG-F01).

***Conflict of interest statement***. None declared.
